# Noninvasive Optical Measurements of Dynamic Cerebral Autoregulation by Inducing Oscillatory Cerebral Hemodynamics

**DOI:** 10.3389/fneur.2021.745987

**Published:** 2021-11-16

**Authors:** Thao Pham, Cristianne Fernandez, Giles Blaney, Kristen Tgavalekos, Angelo Sassaroli, Xuemei Cai, Steve Bibu, Joshua Kornbluth, Sergio Fantini

**Affiliations:** ^1^Department of Biomedical Engineering, Tufts University, Medford, MA, United States; ^2^Department of Neurology, Tufts University School of Medicine, Boston, MA, United States

**Keywords:** cerebral autoregulation, cerebral blood flow, near-infrared spectroscopy, frequency-domain, coherent hemodynamics, neurocritical care, brain, oscillations

## Abstract

**Objective:** Cerebral autoregulation limits the variability of cerebral blood flow (CBF) in the presence of systemic arterial blood pressure (ABP) changes. Monitoring cerebral autoregulation is important in the Neurocritical Care Unit (NCCU) to assess cerebral health. Here, our goal is to identify optimal frequency-domain near-infrared spectroscopy (FD-NIRS) parameters and apply a hemodynamic model of coherent hemodynamics spectroscopy (CHS) to assess cerebral autoregulation in healthy adult subjects and NCCU patients.

**Methods:** In five healthy subjects and three NCCU patients, ABP oscillations at a frequency around 0.065 Hz were induced by cyclic inflation-deflation of pneumatic thigh cuffs. Transfer function analysis based on wavelet transform was performed to measure dynamic relationships between ABP and oscillations in oxy- (*O*), deoxy- (*D*), and total- (*T*) hemoglobin concentrations measured with different FD-NIRS methods. In healthy subjects, we also obtained the dynamic CBF-ABP relationship by using FD-NIRS measurements and the CHS model. In healthy subjects, an interval of hypercapnia was performed to induce cerebral autoregulation impairment. In NCCU patients, the optical measurements of autoregulation were linked to individual clinical diagnoses.

**Results:** In healthy subjects, hypercapnia leads to a more negative phase difference of both *O* and *D* oscillations vs. ABP oscillations, which are consistent across different FD-NIRS methods and are highly correlated with a more negative phase difference CBF vs. ABP. In the NCCU, a less negative phase difference of *D* vs. ABP was observed in one patient as compared to two others, indicating a better autoregulation in that patient.

**Conclusions:** Non-invasive optical measurements of induced phase difference between *D* and ABP show the strongest sensitivity to cerebral autoregulation. The results from healthy subjects also show that the CHS model, in combination with FD-NIRS, can be applied to measure the CBF-ABP dynamics for a better direct measurement of cerebral autoregulation.

## 1. Introduction

Cerebral autoregulation is a homeostatic feedback mechanism that maintains stable cerebral blood flow (CBF) despite moderate changes in arterial blood pressure (ABP). This mechanism utilizes the arteries and arterioles of the brain that can dilate and constrict to regulate CBF and limit its variability. In the Neurocritical Care Unit (NCCU), measurements of cerebral autoregulation in patients with traumatic brain injury can help the diagnosis and monitoring of pathological conditions to improve patient care and to prevent further injury to the brain (1). In fact, impaired cerebral autoregulation is linked to poor clinical outcomes in a variety of conditions such as subarachnoid hemorrhage, stroke, traumatic brain injury, etc. (2–4).

Early studies refer to cerebral autoregulation as a static phenomenon described by a non-linear curve with a characteristic CBF plateau in a range of ABP in which cerebral autoregulation is active (5). With the use of transcranial Doppler ultrasound (TCD) for non-invasive and rapid measurements of CBF, dynamic cerebral autoregulation was introduced by investigating CBF transients in response to dynamic ABP changes (6, 7). One common protocol to assess dynamic autoregulation involves targeting ABP oscillations at low frequencies (<0.2 Hz). Such oscillations can occur spontaneously (8) or can be induced by using various protocols including periodic thigh-cuff inflation (9) and paced breathing (10–12). A transfer function analysis is performed to quantify the relationship between ABP (input, as monitored simultaneously by finger plethysmography) and CBF (output, as monitored by TCD). A more positive phase shift between CBF vs. ABP (i.e., a faster recovery of CBF in response to ABP changes) is often associated with an effective cerebral autoregulation (8, 10, 11, 13). This technique has been applied in the clinical setting to assess cerebral autoregulation in patients with intracranial hypertension (14), traumatic brain injury (15), acute ischemic stroke (16, 17), carotid artery occlusive disease (10), intracranial hemorrhage (4), etc. It was shown that a higher correlation coefficient (15) and a smaller phase difference (16) between CBF and ABP implies worsening autoregulation in those patients. Although TCD together with ABP measurements have been widely used for autoregulation assessment in various population, TCD has its limitations as it cannot measure microvascular and localized changes in CBF.

Oscillations in blood flow and blood volume have individual effects on the oscillations of hemoglobin concentrations that can be sensed by near-infrared spectroscopy (NIRS). Specifically, NIRS is an optical technique that can measure cerebral changes in oxy-, deoxy-, and total hemoglobin concentrations [*O*(*t*), *D*(*t*), and *T*(*t*), respectively]. As compared to TCD, NIRS is sensitive to more local changes in hemodynamics in different compartments of the microvasculature. NIRS can provide measurements with a spatial resolution of less than 4 cm spatially and less than 2 cm in depth (18), thus realizing a better self-contained and spatially congruent technology for local CBF and autoregulation assessment (11). Spontaneous and induced oscillations in hemoglobin concentrations measured by NIRS have been shown to be sensitive to cerebral autoregulation in literature. For instance, studies have reported dynamic cerebral autoregulation measured indirectly through the dynamic relationship between *D* and *O* (10, 19, 20), between *O* and ABP (11, 21–24), between *D* and ABP (10), between *T* and ABP (25), and between cerebral tissue saturation (the ratio of *O* to *T*) and ABP (26, 27). However, there is no clear evidence of which relative dynamic relationship between hemoglobin concentrations and blood pressure is the most sensitive to cerebral autoregulation. This could possibly be due to the issue of NIRS measurements as they are not solely sensitive to CBF but in fact a combination and interplay of CBF, cerebral blood volume (CBV), and cerebral metabolic rate of oxygen (CMRO_2_). As a result, it is highly relevant to translate NIRS measurements into underlying physiological processes, especially to more correct and direct measurements of CBF dynamics. This can be achieved by using a hemodynamic model for coherent hemodynamics spectroscopy (CHS) (28) that converts frequency-domain (FD) NIRS measurements into a relative CBF changes [cbf(*t*)]. This model takes into account the effects of CBV changes and blood transit times in the capillary and venous compartments in the measurements of hemoglobin concentrations (29, 30).

One well-known issue with non-invasive cerebral NIRS using continuous-wave (CW) instruments is the contribution to the optical signal from extra cerebral hemodynamics in superficial tissue (scalp) and in the skull. The most common setup for this kind of measurement is using intensity data in a single-distance (SD) configuration, which consists of one source and one detector separated by a set distance. Single-slope (SS) methods, based on either a single source and multiple detectors or a single detector and multiple sources have been introduced to improve the quality of optical measurements of tissue saturation and to reduce sensitivity to superficial tissue (31, 32). Another approach based on a special configuration of two sources and two detectors was proposed in frequency-domain (FD) spectroscopy for self-calibrating absolute measurements of optical properties in diffuse media (33). This method was implemented in commercial CW-NIRS tissue oximeters (34, 35). More recently, this special arrangement was revisited for enhanced depth discrimination on the basis of separate intensity or phase measurements in FD-NIRS (and named dual-slope (DS) method in this context) (18, 36), or the moments of the photon time of flight distribution in time-domain (TD) NIRS (37). Here, we consider separate intensity (I) and phase (ϕ) measurements obtained with FD-NIRS in SD, SS, and DS configurations. Based on previous studies (18, 38), slope measurements (SS, DS) were shown to be more sensitive to cerebral hemodynamics than SD data, and ϕ data were shown to be more sensitive to the brain than I data. Furthermore, from a practical viewpoint, the DS method also provides a benefit of being largely insensitive to instrumental drifts and optical coupling effects (36). We have demonstrated the potential of NIRS-CHS to measure cerebral autoregulation in healthy subjects in a protocol of rapid step changes in ABP (39). While in our previous study only CW measurements were used (39), here we further explore the potentiality of FD measurements for assessment of dynamic cerebral autoregulation in healthy controls and in the clinical settings.

The scope of this work is to first validate our protocol of induced cerebral hemodynamic oscillations to measure cerebral autoregulation in controlled healthy subjects and in NCCU patients. In the sample of healthy subjects, we first targeted the blood-pressure-induced hemoglobin oscillations (i.e., *O*, *D*, and *T* oscillations) at about 0.065 Hz that are most sensitive to cerebral autoregulation impairment by hypercapnia in healthy subjects. Hypercapnia, a state of elevated arterial CO_2_ concentration above normal levels (normocapnia), causes vasodilation of arterioles and, thus, an increase in CBF (40) and a decrease in cerebral autoregulation capacity (6, 41, 42). In the sample of NCCU patients, the results from healthy subjects were used to guide the clinical interpretation of three patients. We aim to demonstrate the feasibility of using these optical measurements with a protocol of blood-pressure-induced hemodynamic oscillations to monitor cerebral autoregulation efficiency in patients with brain injuries. The second goal of this study is to propose the applicability of the CHS model to provide optical measurements of CBF-ABP dynamics, which is more directly related to the cerebral autoregulation efficiency than NIRS measurements of *O*, *D*, and *T*, without any model. Finally, we aimed at investigating these results with different kinds of FD-NIRS methods (SD, SS, DS with either I or ϕ data) that may feature different relative sensitivities to cerebral vs. extracerebral hemodynamics, thus helping to address the issue of extracerebral tissue contamination in NIRS signals.

## 2. Materials and Methods

### 2.1. Human Subjects

Eight subjects participated in the two studies: five healthy subjects (subjects 1–5; one female, four males, age range: 23–33 yr) and three NCCU patients (patients 1–3; one female, two males, age range: 17–67 yr). The NCCU patients were recruited from Tufts Medical Center, and a summary of patient demographic information can be found in Table 1. Both studies were approved by the Tufts University Institutional Review Board and all participants signed an informed consent prior to the experiment. For the NCCU patients, patient 1 participated on 3 days, patient 2 on 2 days, and patient 3 on 1 day. Each day, the measurement session was repeated three times. We report the results for a single day and measurement session, selected on the basis of low motion artifacts, high signal-to-noise ratio of the optical data, and maximum extent of induced oscillations in ABP and cerebral hemodynamics.

**Table 1:** Summary of patient information from neurocritical care unit.

**Patient**	**Age**	**Sex**	**Cuff Pressure**	**ICP**	**MAP**	**CPP**	**Clinical diagnosis**
			**(mmHg)**	**(mmHg)**	**(mmHg)**	**(mmHg)**	
1	61	M	180	12 [11,14]	82 [80,84]	69 [66,73]	Left basal ganglia hemorrhage
							and parietal area, cerebral
							edema, ventricular effacement
2	67	M	150	0 [•1,1]	81 [79,85]	81 [78,85]	Isolated IVH of unclear etiology
							tissue injury, Hydrocephalus
3	17	F	170	10 [10,11]	73 [68,77]	63 [58,67]	Left occipital AVM with IVH,

*Values are reported as median [25%, 75% quartiles] over the entire experimental time trace.*

*M, Male; F, Female; ICP, Intracranial pressure; MAP, Mean arterial pressure; CPP, Cerebral perfusion pressure; IVH, Intraventricular Hemorrhage; AVM, Arteriovenous Malformation*.

### 2.2. Data Acquisition and Experimental Protocol

**Figure 1:**
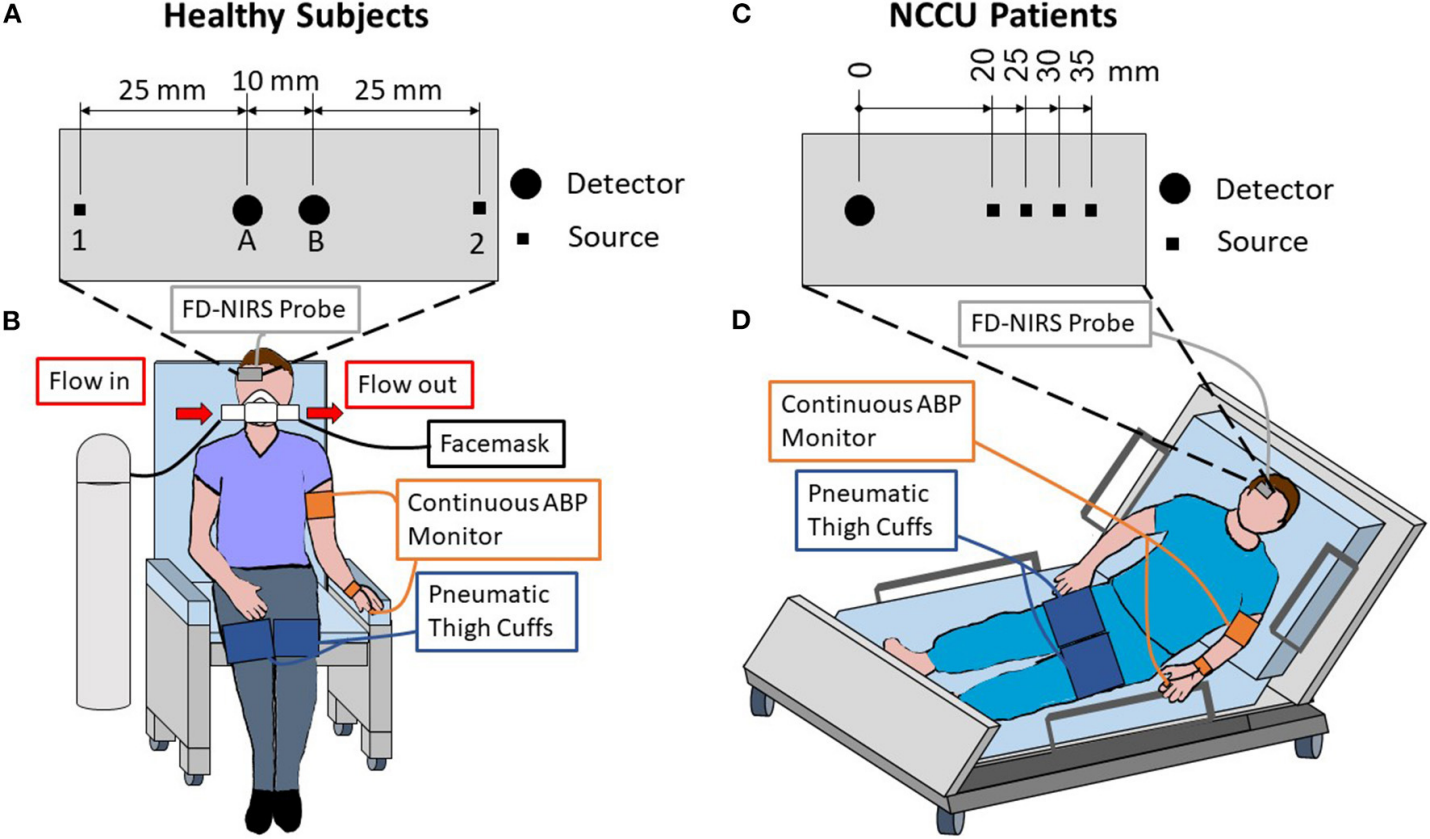
Experimental setup and schematic diagrams of the optical probes used for healthy subjects **(A,B)** and patients in the neurocritical care unit (NCCU; **C,D**). For the healthy subjects, the optical probe was placed on the right side of the forehead and included two sources [1 & 2] and two detectors [A & B] that are symmetrical about the midline of the two sources **(A)**. The probe featured two single-distance (SD) measurements at 35 mm source-detector distances [1B & 2A], two single-slope (SS) measurements with 25 and 35 mm distances [1AB & 2BA], and one dual-slope (DS) measurement with two 25 mm and two 35 mm distances [1AB2]. For the NCCU patients, the optical probe was placed on the right side of the forehead, it featured four source-detector distances of 20, 25, 30, and 35 mm **(C)** in a multi-distance configuration. Both setups included an optical probe, finger plethysmograph to monitor arterial blood pressure (ABP), and pneumatic thigh cuffs wrapped around both thighs **(B,D)**. During the healthy subject experiment **(B)**, a facemask was worn to breath either medical air or 5% CO_2_ mixed with 21% O_2_ and balanced N_2_.

Figure 1 shows the setups for the experiments on healthy subjects (Figures 1A,B) and on NCCU patients (Figures 1C,D). In the healthy subjects, the FD-NIRS measurements were performed using a commercial FD-NIRS instrument (Imagent, ISS Inc., Champaign, IL; wavelengths: 690 and 830 nm; modulation frequency: 140.625 MHz) operating at a data acquisition rate of 9.93 Hz. An optical probe was placed on the right side of the subject's forehead. This probe consisted of a linear array of two source fiber pairs and two detector fiber bundles that are symmetrical about the midline between the two sources. This configuration allows for two SD measurements at 35 mm source-detector distances, two SS measurements at source-detector distances of 25 and 35 mm, and one DS measurement with two sets of 25 and 35 mm source-detector distances (Figure 1A). In the NCCU, FD-NIRS measurements were done using a second commercial system (OxiplexTs, ISS Inc., Champaign, IL; wavelengths: 690 and 830 nm; modulation frequency: 110 MHz) operating at a data acquisition rate of 12.5 Hz. The optical probe was also placed on the right side of the patient's forehead. The probe consisted of one detector fiber bundle and four separate source fiber pairs, with source-detector distances of 20, 25, 30, and 35 mm (Figure 1C). The optical system was calibrated using a phantom of known optical properties, allowing for absolute measurements of tissue optical properties.

In both experiments on healthy subjects and in the NCCU, two pneumatic thigh cuffs were wrapped around the subject's thighs and were connected to an automatic cuff inflation system (E-20 Rapid Cuff Inflation System, D.E. Hokanson, Inc., Bellevue, WA). The air pressure in the thigh cuffs was monitored by a digital manometer (Series 626 Pressure Transmitter, Dwyer Instruments, Inc., Michigan City, IN). The maximum pressure was set to be above systolic blood pressure, specifically to 200 mmHg for healthy subjects, and between 150 and 180 mmHg (reported in Table 1) for the NCCU patients. This value was dependent on individual blood pressure and physician's recommendation. Inflation of the pneumatic thigh cuffs was done smoothly to maximum pressure and took approximately 1 s for healthy subjects and approximately 4 s for NCCU subjects. Previous work (not reported) has shown no effect on results due to inflation times.

In the experiment on healthy subjects, the subject breathed through a facemask (AFT25, BIOPAC Systems, Inc., Goleta, CA) medical air (21% O_2_, 79% N_2_) for 7 min (normocapnic baseline), 5% CO_2_ mixed with 21% O_2_ and balanced N_2_ (5% CO_2_, 21% O_2_, 74% N_2_) for 3 min (hypercapnic interval), and then medical air for 5 min of recovery. The setup for the normocapnia-hypercapnia experiment on healthy subjects was described elsewhere in detail (43). An end-tidal carbon dioxide pressure (PETCO2) signal was collected by using an infrared-based CO_2_ monitor module (CO2100C, BIOPAC Systems, Inc., Goleta, CA) connected to the facemask. The thigh cuff oscillations were performed at a frequency of 0.066 Hz for 1.5 min during the normocapnic baseline and during the second half of the hypercapnia interval. The experimental setup for healthy subjects is shown in Figure 1B. In the NCCU, the experimental protocol consisted of 5 min of baseline, 2 min of thigh cuff oscillations at a frequency of 0.063 Hz, and 5 min of recovery (44). The patients were assumed to be in a normocapnic state as they were either independently breathing room air (patient 2) or on ventilator support (patients 1 and 3). The experimental setup for NCCU patients is shown in Figure 1D.

In all the experiments, continuous ABP was monitored with a finger plethysmography system (NIBP100D, BIOPAC Systems, Goleta, CA). Analog outputs of the ABP monitor, the pneumatic thigh cuff manometer, and the PETCO2 monitor (in the case of healthy subject experiments) were fed to auxiliary inputs of the FD-NIRS instrument for concurrent recordings with the optical data at the same acquisition rate. In the NCCU, intracranial pressure (ICP) from an invasive ICP probe was recorded continuously during each experiment via an Philips Intellivue monitor (Philips Medical Systems, Eindhoven, the Netherlands) and synced to the optical data. The mean arterial pressure (MAP) was calculated from the non-invasive ABP measurements as a weighted average of systolic and diastolic ABP as MAP = [systolic blood pressure+(2× diastolic blood pressure)]/3. These two metrics were used to calculate cerebral perfusion pressure (CPP), which is computed as CPP = MAP − ICP for every time point. Table 1 reports the median value over the experiment with their respective 25% and 75% quartiles for ICP, MAP, and CPP.

### 2.3. Data Processing

#### 2.3.1. Measurements of Absolute and Relative Hemoglobin Concentrations

During the initial baseline periods, average absolute oxy-, deoxy-, and total-hemoglobin concentrations (*O*_0_, *D*_0_, and *T*_0_ = *O*_0_ + *D*_0_, respectively, with subscript “0” indicating average baseline values) were computed. Specifically, these absolute hemoglobin concentration values were obtained from average baseline absorption coefficients (μ*a*_,0_) at two wavelengths (690 and 830 nm) by using known extinction coefficients (45) and an assumed water content of 70% by volume (46). Tissue optical properties at baseline, namely μ*a*_,0_ and the reduced scattering coefficient ($\mu_{s,0}^{\prime }$), were obtained at the two wavelengths using either the self-calibrating method (33) on data from healthy subjects or the calibrated multi-distance method (31) on data from the NCCU patients. Both methods were applied with an iterative approach on the NIRS intensity I and phase ϕ data collected at baseline. A full description of this iterative technique can be found in (47).

Relative changes in hemoglobin concentrations with respect to baseline [Δ*O*(*t*), Δ*D*(*t*), Δ*T*(*t*) = Δ*O*(*t*) + Δ*D*(*t*)] were obtained from relative changes in absorption, Δμ*a*(*t*), at two wavelengths. Here, we calculated Δμ*a*(*t*) by using three different configurations (SD, SS, and DS) and two data types (I and ϕ data). SDI and SDϕ measurements refer to the I and ϕ data, respectively, collected by a single source-detector pair. SSI and SSϕ refer to the linear dependence of an I-based function ($\ln[\frac{\rho^{2}I}{\sqrt{3\mu_{a}\mu_{s}^{\prime }}+1/\rho }]$) and ϕ on source-detector distance ρ, respectively (43). Δμ*a* from SDI and SDϕ were found by using the differential pathlength factors (DPF: DPF_I_ for I and DPF_ϕ_ for ϕ), and from SSI and SSϕ by using the differential slope factors (DSF: DSF_I_ for I and DSF_ϕ_ for ϕ). The expressions of DPF and DSF were described in detail elsewhere (18), and require the measurements of optical properties at baseline μ*a*_,0_ and $\mu_{s,0}^{\prime }$. DS measurements for I or ϕ can be obtained by taking the average of the two symmetrical SS measurements. With the probe configuration used in the two experiments (Figure 1), we reported measurements from two SDI and two SDϕ at 35 mm source-detector distances, two SSI at 25 and 35 mm source-detector distances, one DSI, and one DSϕ for healthy subjects; and measurements from one SDI, one SDϕ at 35 mm, and one SSI measurement at 25 and 35 mm source-detector distances for NCCU patients. SSϕ measurements were not reported in this study due to a poor signal-to-noise ratio (18).

**Figure 2:**
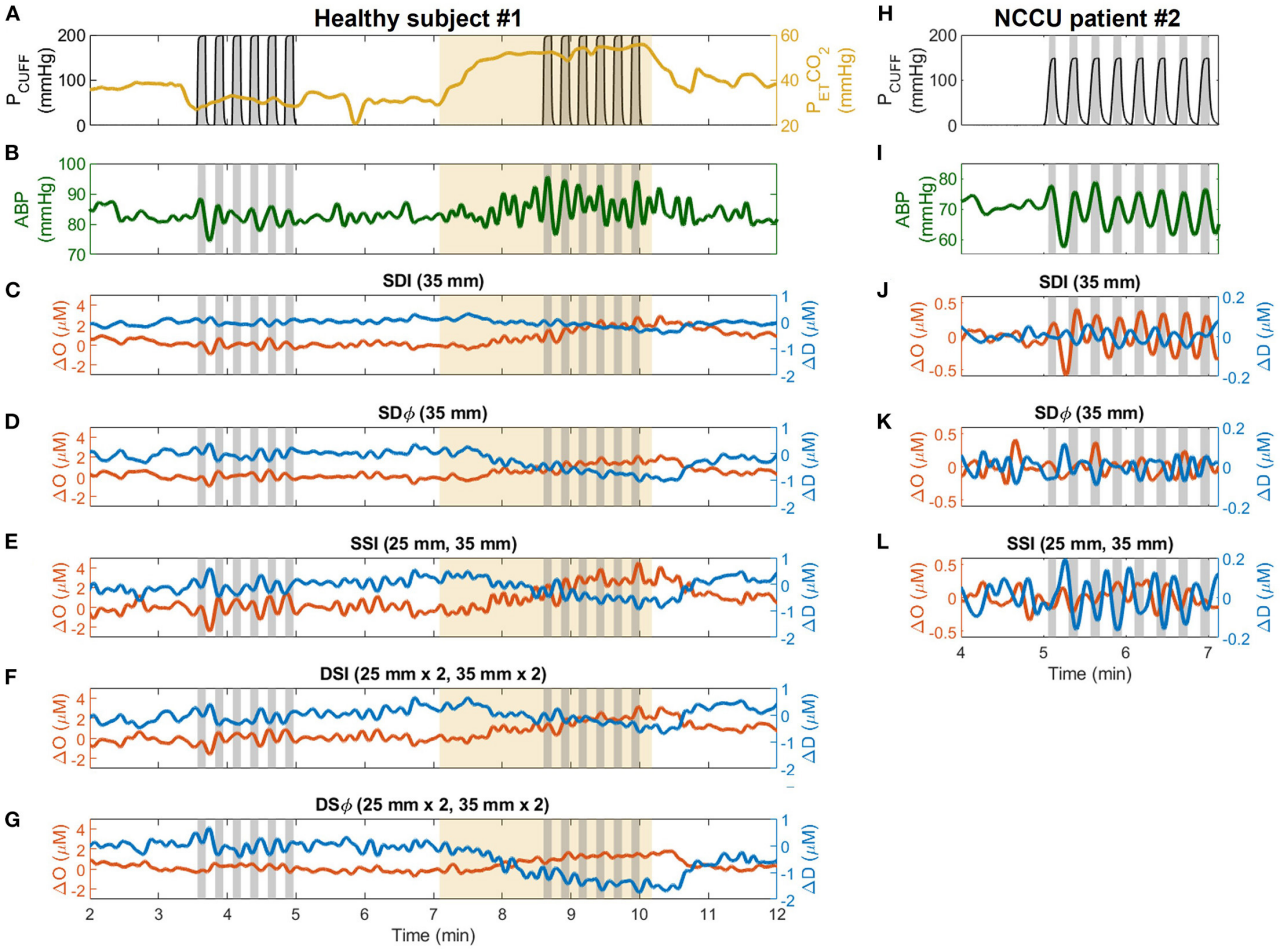
Representative time traces collected during the experiment on healthy subjects (subject 1, *left* column) and patients in the neurocritical care unit (NCCU) (patient 2, *right* column). For the healthy subject, signals are shown for pneumatic thigh cuff pressure (P_CUFF_, *black*, **A**), end-tidal carbon dioxide pressure (PETCO2, *yellow*, **A**), arterial blood pressure (ABP, *green*, **B**), and changes in oxyhemoglobin (Δ*O*, *red*) and deoxyhemoglobin (Δ*D*, *blue*) measured with single-distance intensity (SDI 2A, 35 mm, **C**), single-distance phase (SDϕ 2A, 35 mm, **D**), single-slope intensity (SSI 1AB, 25 and 35 mm, **E**), dual-slope intensity (DSI 1AB2, 25 and 35 mm, **F**), and dual-slope phase (DSϕ 1AB2, 25 and 35 mm, **G**). For the NCCU patient, signals are shown for P_CUFF_ **(H)**, ABP **(I)**, and for Δ*O* and Δ*D* measured with SDI (35 mm, **J**), SDϕ (35 mm, **K**), and SSI (25 and 35 mm, **L**). Gray shaded areas indicate the cuff inflation periods (from start of inflation to start of deflation). Thigh cuffs were inflated and deflated at a frequency of 0.066 Hz for healthy subjects and at 0.063 Hz for NCCU patients. Yellow shaded areas indicate hypercapnic interval. ABP and optical signal traces are lowpass filtered to 0.1 Hz.

Figure 2 shows typical experimental time traces for a representative healthy subject (subject 1) and a representative NCCU patient (patient 2). The signals include: the recorded pneumatic thigh cuff pressure (P_CUFF_), PETCO2 (for the healthy subject), ABP, and time traces of Δ*O* and Δ*D* obtained with different data analysis methods.

#### 2.3.2. Wavelet Analysis of Phasor Ratios

We performed transfer function analysis using Wavelet coherence and phasor analysis to determine the phase differences and amplitude ratios of the coherent hemodynamic oscillations (i.e., oscillations in *O*, *D*, *T*) vs. ABP oscillations within the interval of the thigh cuff inflation-deflation oscillations. The processing steps were the same for both healthy subjects and NCCU patients. These analyses were described in references (20, 38) and are summarized here.

Briefly, a continuous wavelet transform with complex Morlet mother wavelet was used to get two-dimensional phasor maps of **D**(*t*, ω), **O**(*t*, ω), **T**(*t*, ω), and **ABP**(*t*, ω), as functions of time (*t*) and frequency (ω). Note that we use bold-face notations to indicate phasor values. From these phasor maps, we computed the phasor ratio maps of $\frac{\text{D}\left(t,\omega \right)}{\text{ABP}\left(t,\omega \right)}$, $\frac{\text{O}\left(t,\omega \right)}{\text{ABP}\left(t,\omega \right)}$, and $\frac{\text{T}\left(t,\omega \right)}{\text{ABP}\left(t,\omega \right)}$. The phasor ratio maps refer to the dynamic relationship between oscillations of hemoglobin concentrations and ABP(*t*) at time *t* and angular frequency ω. Along with the phasor ratio maps, two-dimensional wavelet coherence maps between two signals (*O* vs. ABP, *D* vs. ABP, and *T* vs. ABP) were also computed. A measure of coherence defines coupling between one signal (ABP) and another [*O*(*t*), *D*(*t*), or *T*(*t*)]. A high coherence value (closer to 1) can be used to ensure the validity of phase and amplitude estimations of the phasor ratios (8, 13). We applied a map of coherence threshold values on each corresponding coherence map, and only considered the corresponding time-frequency pixels in the phasor ratio maps with coherence that passed the threshold (significantly high coherence) for further analysis. The coherence threshold map was generated from random surrogate data (48, 49) at a significance level of α = 0.05. For the phasor ratio $\frac{\text{D}\left(t,\omega \right)}{\text{O}\left(t,\omega \right)}$, we used the regions in the time-frequency space with significantly high coherence for both *O* vs. ABP and *D* vs. ABP.

After applying the coherence threshold, we only considered the average phasor ratios **D**/**ABP**, **O**/**ABP**, **T**/**ABP**, and **D**/**O** with significantly high coherence within the time intervals of the thigh cuff oscillation and a frequency band centered at the central frequency of the induced oscillation. The central frequency was 0.066 Hz for the healthy subject data and 0.063 Hz for the NCCU patient data. The bandwidth of the frequency band was determined by the half power bandwidth of a simulated test sinusoidal signal, which resulted in six frequency bands in the range of 0.059–0.079 Hz for the healthy subject data, and five frequency bands in the range of 0.055–0.074 Hz for the NCCU patient data. Further criteria required to include an individual phasor sample in the average were that the region of interest consisted of continuous coherence longer than one period of the oscillation at the central frequency and at least two continuous frequency bands (where one is the central frequency). The argument of the phasor ratio represents the phase difference between the two phasors, and the magnitude represents the amplitude ratio of the two phasors. We used standard statistics and circular statistics (50) for the calculation of the mean and standard deviation of amplitudes and phase angles, respectively, within the induced oscillation interval with significantly high coherence. Standard errors of the measurements were calculated by dividing the standard deviations by the square root of number of independent observations, which was taken as the number of periods at the induced oscillation that passed the coherence threshold. This amplitude and phase analysis was applied to data collected with all the different analysis methods: SDI, SDϕ, SSI, DSI, and DSϕ for healthy subjects; SDI, SDϕ, and SSI for NCCU patients.

#### 2.3.3. Hemodynamic Model to Determine the Relative Cerebral Blood Flow and Arterial Blood Pressure Dynamics

In the sample of healthy subjects, we applied the dynamic hemodynamic model of CHS introduced by Fantini (28) to calculate dynamic relationship between CBF and ABP. Specifically, the two-dimensional map of the phasor ratio $\frac{\text{cbf}\left(t,\omega \right)}{\text{abp}\left(t,\omega \right)}$ were obtained from the phasor ratio maps of $\frac{\text{D}\left(t,\omega \right)}{\text{ABP}\left(t,\omega \right)}$, $\frac{\text{O}\left(t,\omega \right)}{\text{ABP}\left(t,\omega \right)}$, and $\frac{\text{T}\left(t,\omega \right)}{\text{ABP}\left(t,\omega \right)}$ with significantly high coherence. Here, cbf and abp refer to normalized changes in CBF (in units of mL_blood_/100 g_tissue_/min) and ABP, respectively, with respect to baseline values. The computation of the phasor ratio $\frac{\text{cbf}\left(t,\omega \right)}{\text{abp}\left(t,\omega \right)}$ by using the frequency-domain CHS model is described in detailed in the Supplementary Material. Briefly, the CHS model provides an analytical way to describe how NIRS measurements of hemoglobin concentrations *O*, *D*, and *T* are related to the effects of changes in blood volume in three microvascular compartments (arterial, capillary, venous) and the effects of changes in blood flow and metabolic rate of oxygen in the capillary and venous compartments. Under the assumption of negligible changes in CMRO_2_ during the dynamic oscillations, the CHS model can provide measurements of cbf from NIRS measurements of *O*(*t*) and *D*(*t*). Additional assumptions include negligible changes in capillary (*c*) blood volume cbv^(*c*)^(*t*) = 0 and equal arterial (*a*)-to-venous (*v*) dynamic CBV ratio $\frac{{\text{cbv}}^{\left(a\right)}}{{\text{cbv}}^{\left(v\right)}}=1$. We assumed values for CHS model parameters such as capillary baseline CBV fraction $\frac{F^{\left(c\right)}{\text{CBV}}_{0}^{\left(c\right)}}{{\text{CBV}}_{0}}=0.4$, baseline arterial and venous CBV ratios as $\frac{{\text{CBV}}_{0}^{\left(a\right)}}{{\text{CBV}}_{0}}=\frac{{\text{CBV}}_{0}^{\left(v\right)}}{{\text{CBV}}_{0}}=0.3$, capillary blood transit time *t*(*c*) = 1 s, venous blood transit time *t*(*v*) = 5 s, and rate constant of oxygen diffusion α = 0.8 *s*^−1^ based on values from healthy human subjects (30). The calculation also requires the input of absolute baseline total-hemoglobin concentration *T*_0_. Using the same method and criteria as described in section 2.3.2, we computed the average phasor ratio **cbf**/**abp** within the time interval of the thigh cuff oscillation and a frequency band centered at the central frequency of the induced oscillation.

### 2.4. Statistical Analysis

Statistical tests were used to assess the differences between normocapnia and hypercapnia for phase differences and amplitude ratios of **D** vs. **ABP**, **O** vs. **ABP**, **T** vs. **ABP**, **cbf** vs. **abp**, and **D** vs. **O**. Specifically, the one-sample test for angular mean (50) was applied to test if the mean paired phase difference values during hypercapnia and normocapnia (hypercapnia − normocapnia) is significantly less than 0° (one-tailed test). A paired *t*-test on a linear scale was applied on the amplitude ratios to test if the mean paired difference between the amplitude ratio values during normocapnia and hypercapnia is significantly different from 0 (two-tailed test). These statistical tests assume that the phase values follow a von Mises distribution (50), and the amplitude ratio values follow a normal distribution. A value of *p* < 0.05 was considered as significant.

## 3. Results

### 3.1. Phase and Amplitude Relations in Healthy Controls: Normocapnia vs. Hypercapnia

**Figure 3:**
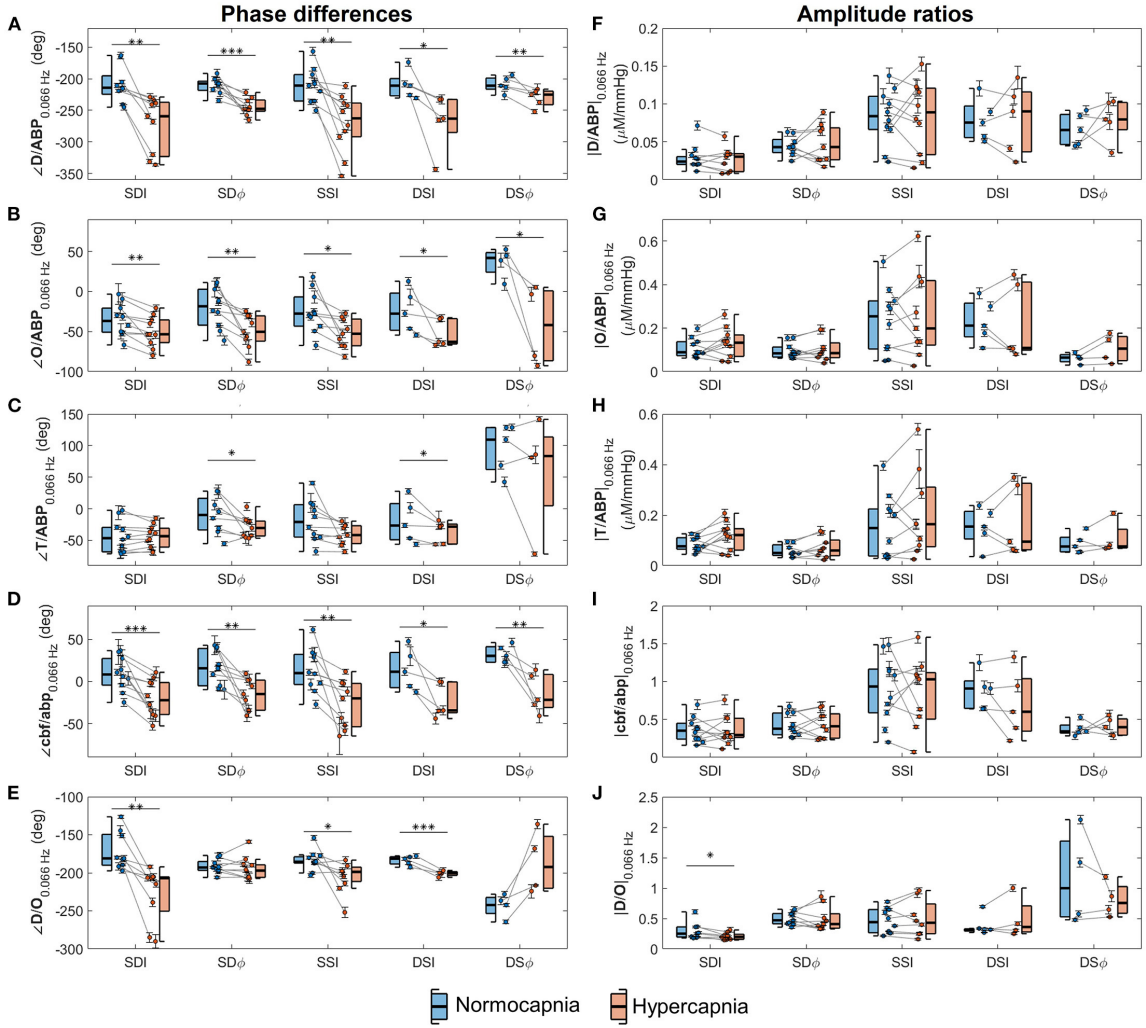
Box plots depicting average phase differences (*left* column) and amplitude ratios (*right* column) of phasors of deoxyhemoglobin (**D**) vs. phasors of arterial blood pressure (**ABP**) (panels **A,F**), oxyhemoglobin (**O**) vs. **ABP** (panels **B,G**), total hemoglobin (**T**) vs. **ABP** (panels **C,H**), normalized cerebral blood flow (**cbf**) vs. normalized arterial blood pressure (**abp**) (panels **D,I**), and **D** vs. **O** (panels **E,J**) at 0.066 Hz. Boxes are shown for normocapnia (*blue*) and hypercapnia (*red*). On each box, the central line is the median, the edges are the 25 and 75% quartiles, and the whiskers indicate the most extreme data points excluding outliers. Circles indicate individual measurements, and error bars represent the standard errors of the measurements. Each *gray* line connects normocapnic and hypercapnic data points from the same measurement session. Horizontal lines (*black*) indicate comparison between normocapnia and hypercapnia by means of statistical tests and corresponding *p*-values are denoted (*p < 0.05; **p < 0.01; ***p < 0.001).

From five healthy subjects, hypercapnia caused a significant increase in PETCO2 from 37 ± 2 mmHg to 54 ± 1 mmHg (mean ± standard error, paired *t*-test *p* = 0.001). Figure 3 displays box plots showing phase and amplitude measurements of **D**/**ABP**, **O**/**ABP**, **T**/**ABP**, **cbf**/**abp**, and **D**/**O** obtained from five healthy subjects during normocapnia and hypercapnia for different FD-NIRS methods (SDI, SDϕ, SSI, DSI, and DSϕ). The median values and the respective [25%, 75%] quartiles of the phase values for normocapnia and hypercapnia are reported in Table 2. Note that for every subject from this data set, two measurements of SD and two measurements of SS were reported.

**Table 2:** Values of the phase differences of phasors of deoxyhemoglobin (**D**) vs. arterial blood pressure (**ABP**), oxyhemoglobin (**O**) vs. **ABP**, total hemoglobin (**T**) vs. **ABP**, normalized cerebral blood flow (**cbf**) vs. normalized arterial blood pressure (**abp**), and **D** vs. **O** during normocapnia and hypercapnia.

		**Phase difference (°)**						
		**Normocapnia (NC)**		**Hypercapnia (HC)**		**HC − NC**	****p**-values**	
∠**D**/**ABP**	SDI	−214	[−225, −195]	−259	[−323, −237]	−50 ± 33	0.003**	(*n* = 8)
	SDϕ	−208	[−219, −204]	−248	[−252, −234]	−34 ± 19	0.0003***	(*n* = 9)
	SSI	−211	[−235, −193]	−263	[−292, −239]	−59 ± 41	0.001**	(*n* = 10)
	DSI	−211	[−227, −200]	−263	[−285, −233]	−56 ± 38	0.02*	(*n* = 5)
	DSϕ	−211	[−217, −199]	−225	[−241, −220]	−21 ± 12	0.005**	(*n* = 5)
∠**O**/**ABP**	SDI	−37	[−51, −21]	−53	[−64, −36]	−14 ± 12	0.001**	(*n* = 10)
	SDϕ	−18	[−42, 3]	−50	[−62, −30]	−33 ± 23	0.002**	(*n* = 9)
	SSI	−28	[−43, −7]	−53	[−68, −34]	−26 ± 26	0.02*	(*n* = 9)
	DSI	−28	[−48, −2]	−63	[−66, −34]	−27 ± 24	0.03*	(*n* = 5)
	DSϕ	42	[24, 49]	−42	[−87, 1]	−78 ± 38	0.01*	(*n* = 4)
∠**T**/**ABP**	SDI	−47	[−68, −30]	−43	[−61, −31]	−1 ± 8	0.4	(*n* = 10)
	SDϕ	−10	[−34, 17]	−30	[−43, −19]	−22 ± 26	0.02*	(*n* = 8)
	SSI	−21	[−45, 7]	−42	[−55, −27]	−17 ± 27	0.09	(*n* = 9)
	DSI	−27	[−49, 8]	−28	[−56, −24]	−17 ± 17	0.04*	(*n* = 5)
	DSϕ	109	[62, 129]	83	[5, 114]	−20 ± 49	0.2	(*n* = 5)
∠**cbf**/**abp**	SDI	8	[−4, 27]	−22	[−39, 1]	−29 ± 19	0.0006**	(*n* = 10)
	SDϕ	16	[−5, 39]	−15	[−34, 2]	−34 ± 26	0.006**	(*n* = 9)
	SSI	10	[−4, 32]	−20	[−54, −2]	−40 ± 34	0.006**	(*n* = 9)
	DSI	12	[−7, 34]	−34	[−37, −1]	−36 ± 24	0.01*	(*n* = 5)
	DSϕ	30	[23, 41]	−22	[−31, 8]	−46 ± 24	0.006**	(*n* = 5)
∠**D**/**O**	SDI	−181	[−190, −149]	−207	[−250, −205]	−44 ± 30	0.004**	(*n* = 8)
	SDϕ	−193	[−197, −185]	−197	[−206, −189]	−1 ± 9	0.4	(*n* = 9)
	SSI	−186	[−187, −179]	−199	[−212, −193]	−26 ± 26	0.03*	(*n* = 8)
	DSI	−181	[−189, −179]	−201	[−203, −199]	−16 ± 5	0.0007***	(*n* = 4)
	DSϕ	−242	[−254, −232]	−192	[−220, −152]	56 ± 31	0.5	(*n* = 4)

Values are reported as median [25%, 75% quartiles] for phase measurements at normocapnia and hypercapnia. The differences in the phase values between hypercapnia and normocapnia are shown as means ± standard deviations. p-values from statistical test on angular means are shown and indicated in red color for significant values; * for p < 0.05; ** for p < 0.01; and *** for p < 0.001.

In general, the results presented by Figure 3 and Table 2 show that the relative phase of **D** vs. **ABP** (Figure 3A), **O** vs. **ABP** (Figure 3B), and **cbf** vs. **abp** (Figure 3D) are sensitive to autoregulation efficiency in healthy subjects across all measurement methods. The amplitude ratios are generally insensitive to autoregulation in all measurement methods and parameters. For the phase relationships, we observe a sensitivity to cerebral dynamic autoregulation for the relative phase of **D** vs. **ABP** (*p* < 0.02), **O** vs. **ABP** (*p* < 0.03), and **cbf** vs. **abp** (*p* < 0.01). Specifically, from normocapnia to hypercapnia, the phase of **D** vs. **ABP** is reduced by ~40° across all the methods, the phase of **O** vs. **ABP** is reduced by ~30°, and the phase of **cbf** vs. **abp** is reduced by ~25°. On the other hand, the relative phase of **T** vs. **ABP** shows significance for only two methods (SDϕ and DSI, *p*= 0.02 and 0.04, respectively) with the others showing no significant phase difference (remaining measurement methods, *p*>0.09) to a hypercapnia-induced change in cerebral autoregulation.

While both ∠**D**/**ABP** and ∠**O**/**ABP** indicate a significant change in response to a change in cerebral autoregulation efficiency in healthy subjects, **D**/**ABP** displayed a larger average change in phase in three of the five methods compared to **O**/**ABP** (Table 2). Statistically, the phase change in ∠**D**/**ABP** between normocapnia and hypercapnia is significantly greater than the phase change in ∠**O**/**ABP** (*p* = 0.02). This suggests that *D* oscillations could be a stronger indicator of dynamic cerebral autoregulation compared to *O* oscillations. Oscillations of *D* and *O* during normocapnia and hypercapnia had a mean phase difference of approximately −180° to −250°, indicating a counterphase relationship. For **D**/**O**, only measurements from I showed a significant reduction in phase from normocapnia to hypercapnia (SDI, SSI, and DSI; *p* < 0.03), while ϕ measurements showed no significant change to a change in cerebral autoregulation efficiency (SDϕ, *p* = 0.4; DSϕ, *p* = 0.5).

Finally, all the measurement methods (SDI, SDϕ, SSI, DSI, DSϕ) are sensitive to cerebral autoregulation changes as measured by ∠**O**/**ABP**, ∠**D**/**ABP**, and ∠**cbf**/**abp**, with *p* ≤ 0.03 for all methods and parameters as shown in Table 2. Statistical tests show no significant difference between DSI and DSϕ in the phase change from normocapnia to hypercapnia for ∠**O**/**ABP**, ∠**D**/**ABP**, and ∠**cbf**/**abp** (*p* > 0.1). Between SDI and SDϕ, only ∠**O**/**ABP** shows a significant difference in the normocapnia-hypercapnia phase changes (*p* = 0.009), while other parameters show no significant difference (*p* > 0.05).

### 3.2. Phase Relations in NCCU Patients

**Figure 4:**
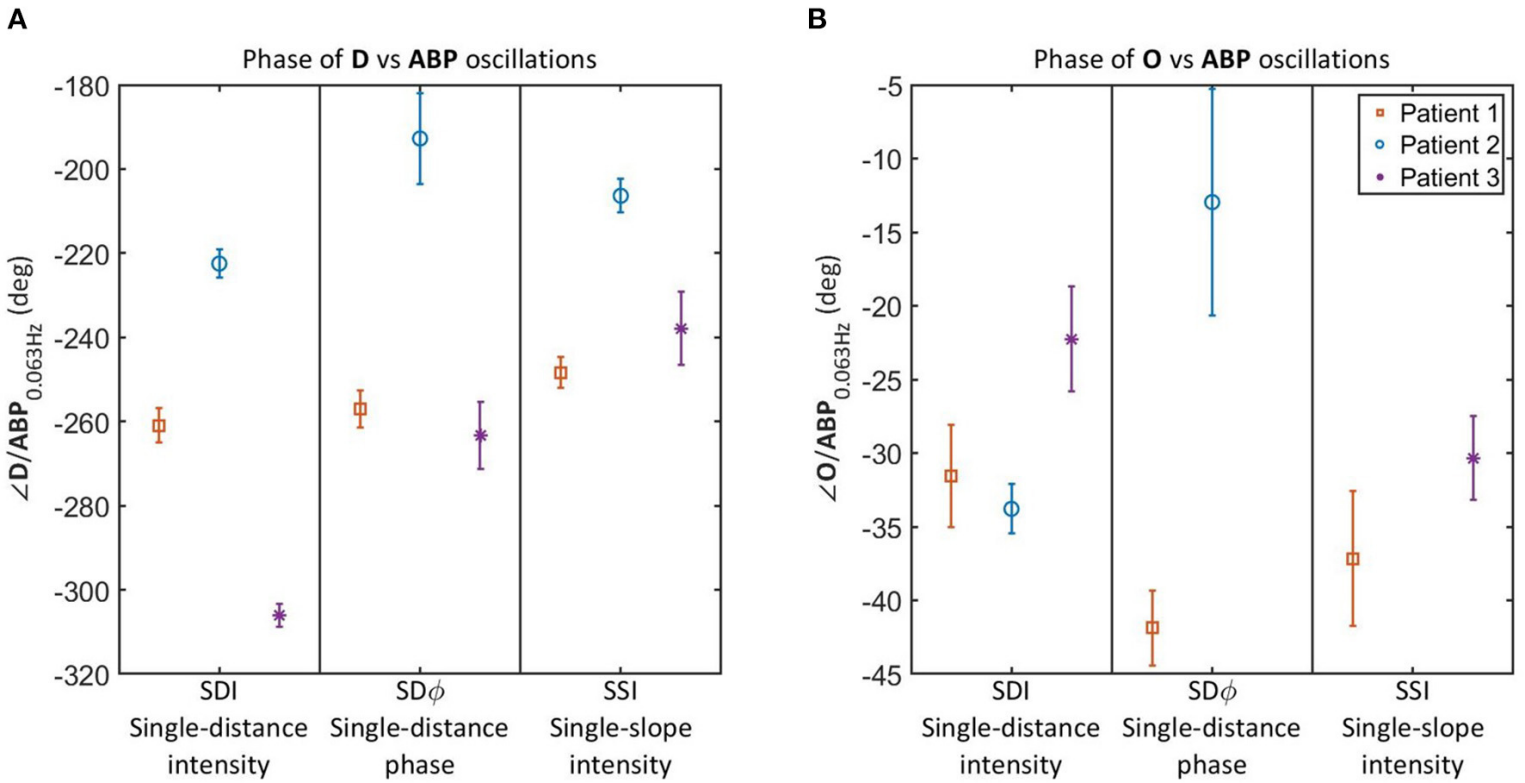
Relative phase of **D** vs. **ABP (A)** and **O** vs. **ABP (B)** for oscillations at 0.063 Hz in 3 NCCU patients measured with single-distance intensity (SDI), single-distance phase (SDϕ), and single-slope intensity (SSI). Color and marker style are consistent for each subject. Error bars represent the standard errors of the measurements. Data for SDϕ in patient 3 and SSI in patient 2 are missing due to a coherence level below the threshold.

Figure 4 reports the relative phase of **D** vs. **ABP** (Figure 4A) and **O** vs. **ABP** (Figure 4B) for the three NCCU patients. Results are shown for SDI 35 mm, SDϕ 35 mm, and SSI 25 and 35 mm. The reason for reporting the relative phases of **D** vs. **ABP** and **O** vs. **ABP** for NCCU patients (Figure 4) is that the results on healthy subjects (Figure 3) showed them to be most sensitive to autoregulation changes, more so than the relative phases of **T** vs. **ABP** and **D** vs. **O**, and any amplitude ratios. We also opted to not report the phase of **cbf** vs. **abp** in NCCU patients because of the need to assume values of hemodynamic parameters (capillary and venous blood transit times, partial blood volume in the arterial, capillary, and venous compartments, etc.) that are expected to be more variable in NCCU patients than in healthy subjects.

It should be noted that the results of Figure 4 allow for a patient by patient comparison, but the number of patients (three) is too small to draw general conclusions. Additionally, we are lacking an independent measurement of cerebral autoregulation in each subject, and cannot make a definite statement on reproducibility among trials in individual patients due to lack of clean data for all three patients. Patients 1 and 3 had high quality data for only 1 day and trial, whereas patient 2 had multiple trials with acceptable data quality in a given day. The inter-trial variability of the relative phase of **D** vs. **ABP** in patient 2 was smaller than the inter-patient differences reported in Figure 4A. However, more work needs to be done to better characterize intra-patient and inter-patient variability in these non-invasive optical measurements of cerebral hemodynamics. Nevertheless, these results allow us to report the technical feasibility of NIRS measurements of cerebral oscillatory hemodynamics that are coherent with ABP. Furthermore, the measurements on healthy subjects during normocapnia and hypercapnia provide a key reference to read and interpret the results observed in the NCCU patients in relation to different levels of cerebral autoregulation.

Results on healthy subjects (Figure 3A) show that an impairment of cerebral autoregulation (during hypercapnia) results in a more negative relative phase of **D** vs. **ABP** as compared to normocapnia. In the NCCU patients (Figure 4A), we observe a less negative phase of **D** vs. **ABP** (with all measurements: SDI, SDϕ, SSI) in patient 2 compared to patients 1 and 3, suggesting a better autoregulation in patient 2. The phase between **O** and **ABP** also became more negative with impaired autoregulation in healthy subjects (as shown in Figure 3B). The results for the relative phase of **O** vs. **ABP** in the NCCU (Figure 4B) were not as clear as those for the relative phase of **D** vs. **ABP**, because of lack of some data (low coherence) for SDϕ patient 3 and SSI patient 2, and opposite results for SDI (more negative phase for patient 2) and SDϕ (less negative phase for patient 2). This result is in line with the smaller impact of autoregulation changes in the phase of **O** vs. **ABP** than **D** vs. **ABP** in the data from healthy subjects. This may be a consequence of incoherent hemodynamics contributions from the arterial compartment in scalp tissue.

## 4. Discussion

This study showed that: (1) relative dynamics of cerebral *O* and *D* vs. ABP dynamics are sensitive to dynamic cerebral autoregulation changes between normocapnia and hypercapnia in healthy subjects, with the relative phase of **D** vs. **ABP** being the most sensitive indicator of cerebral autoregulation efficiency; (2) the relative phase of **D** vs. **ABP** measured in the oscillation protocol can be used in the NCCU to assess the degree of cerebral autoregulation in patients with brain injuries; (3) it is feasible to measure CBF-ABP dynamics by applying the CHS model on the blood-pressure-induced oscillations in cerebral *O* and *D* measured by FD-NIRS in healthy adult subjects; and (4) all the measurement methods (SDI, SDϕ, SSI, DSI, DSϕ) were found to have similar sensitivity to cerebral autoregulation changes for this specific protocol of hemodynamic oscillations.

The results from healthy subjects showed that both blood-pressure-induced *O* and *D* dynamics are sensitive to dynamic cerebral autoregulation, but the phase difference of **D** vs. **ABP** showed stronger sensitivity to the cerebral autoregulation changes between normocapnia and hypercapnia than **O** vs. **ABP**. We have shown that these results were consistent across different measurements within five healthy subjects. These results are somewhat in line with the NCCU patients results that suggest *D* oscillations as more robust than *O* oscillations for the assessment of cerebral autoregulation in the NCCU. Many studies in the literature have used *O* oscillations or the **O**-**ABP** phase difference to assess dynamic cerebral autoregulation, mostly because *O* changes are sensitive to all vascular compartments while *D* changes are more sensitive to the venous compartment (23). On the other hand, the results from NCCU patients in our study are consistent with the results from (10), who reported a significant change in the phase difference of **D** vs. **ABP** and no significant change in the phase difference of **O** vs. **ABP** between healthy controls (i.e., intact cerebral autoregulation) and the affected hemisphere of patients with unicarotid stenosis (i.e., impaired cerebral autoregulation). This observation may result from the fact that *O* and *D* are impacted to a similar extent by blood flow changes, whereas *O* is impacted to a greater extent by blood volume changes than *D*. Specifically, *O* is affected by blood volume changes in all vascular compartments, while *D* is mostly affected by blood volume changes in the capillaries and venous compartment. Our results on healthy subjects have shown that the phase of CBF oscillations is sensitive to cerebral autoregulation efficiency, while CBV (or *T*) oscillations do not exhibit significant changes between normocapnia and hypercapnia. This could explain the observation that *D* oscillations are more sensitive to cerebral autoregulation changes than *O* oscillations. Furthermore, it has been observed that NIRS measurements of task-evoked *O* changes measured non-invasively with NIRS correlate more closely than *D* changes with extracerebral functional magnetic resonance imaging (fMRI) signals (51–53), and that scalp tissue has poorer cerebral autoregulation efficiency than in the brain (54).

The pilot clinical study on NCCU patients demonstrated the proof of concept and technical feasibility of applying a protocol involving induced oscillations in ABP and associated coherent cerebral hemodynamics oscillations in a clinical settings. The non-invasive measurement of these coherent cerebral hemodynamics with FD-NIRS techniques, in conjunction with well-defined criteria for the required levels of coherence with ABP, allows for the measurement of quantities that are sensitive to the degree of dynamic cerebral autoregulation. The representative results reported here on three NCCU patients suggest better cerebral autoregulation conditions for patient 2 compared to patients 1 and 3 based on the relative phase of **D** vs. **ABP**. The values of the relative phase of **D** vs. **ABP** for patient 2 are also within the range observed in healthy subjects (−222° to −192° across methods for patient 2, and −214° to −208° for healthy subjects). This is bolstered by the fact that patient 2 recorded the lowest ICP value and highest CPP value over the time of the trial compared to the other two patients, as reported in Table 1. A higher ICP value is associated with decreased intracranial compliance, which can lead to lower CPP (55). Clinically, this could be a manifestation of poor cerebral autoregulation (4) resulting in reduced CBF (56). Higher ICP values in patients with a traumatic brain injury have previously been shown to be associated with poorer dynamic autoregulation (14), which alludes to the potential for patient 2 to have better autoregulation compared to the other two patients. The clinical diagnosis between patients (Table 1) indicates that patient 2 had no apparent brain injury and had an intraventricular hemorrhage that caused an obstruction of cerebral spinal fluid (CSF) flow. On the other hand, patient 1 had a large hemorrhage in the left frontal and parietal lobes, and patient 3 had a left occipital hemorrhage, both of these causing a larger brain injury than in patient 2. This larger brain damage in patients 1 and 3 as compared to patient 2 can potentially explain the results seen.

Oscillations in cerebral *O* and *D* as measured by FD-NIRS are the result of oscillations in blood flow and blood volume in the investigated tissue. One significant result of this study on healthy subjects is that we were able to show the feasibility of measuring CBF dynamics from *O* and *D* measurements by using the CHS model, and that the results have demonstrated the sensitivity of the **cbf**-**abp** dynamic relationship on cerebral autoregulation differences during normocapnia and hypercapnia in all of the measurement methods with FD-NIRS. We have also shown that the changes in cerebral *T* represent changes in local blood volume that are insensitive to cerebral autoregulation. This is consistent with (10). In this study, we did not report **cbf** vs. **abp** for NCCU patients since the absolute phase values depend on the assumption of parameter values for the CHS model. We also notice that the reported range of normocapnic phase difference of **cbf** vs. **abp** from healthy subjects (about 8° to 30°) is lower than the expected value of 40° (interquartile range of 30°) as measured by TCD for a frequency range of 0.02–0.07 Hz (8). The phase difference of **cbf** vs. **abp** depends on the assumption of model parameters. For instance, an increase in *t*(*c*) from 0.4 to 1 s can cause an increase in the relative phase of **cbf** vs. **abp** by about 10°, and an increase in *t*(*v*) from 3 to 7 s can increase the relative phase by up to 20°. However, we note that the relative phase difference between normocapnia and hypercapnia of **cbf** vs. **abp** is less affected by the parameter values assumptions. For example, this relative phase difference only changes by less than 5° and 1° with an increase in *t*(*c*) from 0.4 to 1 s and *t*(*v*) from 3 to 7 s, respectively. Future studies may include adding induced ABP oscillations at various frequencies (44, 57) or adding a transient change in ABP (30) to find individual values of CHS model parameters. In fact, we have previously demonstrated that the fitting procedure of CHS model at various frequencies to calculate CHS parameters are feasible for applications in the NCCU (44).

The relationship between cerebral **D** and **O** during normocapnia and during impaired autoregulation induced by hypercapnia was also investigated. Many previous studies have considered the phase difference between *D* and *O* oscillations measured by SDI as an indication of cerebral autoregulation (10, 19, 20). In this study, we have shown that different behaviors were observed for **D**/**O** measurements with SDI, SDϕ and DSϕ. Thus, using the dynamic phase relationship between **D** and **O** to interpret cerebral autoregulation may be misleading since the results depend on the measurement method. One possible explanation, at least for the protocol of hypercapnia and normocapnia on healthy subjects considered here, is that **D**/**O** results from the interplay of **O** and **D**, which may feature different sensitivities to hypercapnic-induced vascular hemodynamics in extracerebral and cerebral tissue.

Finally, all the measurement methods (SDI, SDϕ, SSI, DSI, DSϕ) were found to be sensitive to cerebral autoregulation changes as measured by **D**/**ABP** and **cbf**/**abp**. This result may suggest a similar performance of different FD-NIRS measurement methods for sensing autoregulation efficiency in the brain. However, systemic ABP oscillations can induce changes both in blood flow and blood volume of both cerebral and extracerebral tissue. This makes it difficult to interpret the measured quantity without fully understanding the blood volume dynamics in the scalp and the brain. One may notice that the DSϕ measurement of the phase difference of **T** vs. **ABP** at normocapnia is different from other measurement methods, having a positive phase. This may tell us that this measurement is sensitive to different dynamics of blood volume compared to other methods. Finally, a greater sensitivity to deeper tissue (compared to superficial tissue) has been demonstrated for the slope measurements, especially for DSϕ, in a homogeneous tissue setting (18, 38), but the situation can be more complicated in the presence of tissue heterogeneity and different scalp/skull anatomy of the subjects (43). We also stress that the SSϕ data was not reported in both healthy subjects and NCCU patients due to poor signal-to-noise ratio of the phase measurements, particularly in the SS configuration. The application of DSϕ holds promise as it is less affected by noise than SSϕ and largely insensitive to optical couplings and motion artifacts (18).

The results reported in this study are limited by the small sample size of NCCU patients. Due to this, we could not draw any statistical conclusions from the NCCU study but use this data set to show that the collection and processing techniques applied on healthy subjects is applicable outside of the laboratory setting. The major limitation of this approach is that it relies on the need to induce oscillations in blood pressure that have significant coherence with hemodynamic signals. In the NCCU, the thigh cuff occlusions may cause discomfort, and may not be applicable in obese patients. An alternative approach is to use spontaneous oscillations, but the high coherence between hemoglobin concentrations and blood pressure are not usually guaranteed. The second limitation is the requirement of CHS model parameters to calculate **cbf**(ω). In this study, we assumed values for the CHS parameters for healthy controls based on reported range (30). However, for NCCU patients whose physiological conditions are significantly different from healthy subjects, correct values for the model parameters are needed. This usually requires a complicated fitting procedure of CHS model to measurements obtained at multiple induced oscillation frequencies or with a transient change in ABP (30, 44, 57). Finally, subject's position and posture were different between the NCCU patients (lying down) vs. healthy subjects (sitting), which may have affected the variations in CBF dynamics (58, 59). Future studies will focus on the effects of the subject's position and different head of bed angle manipulation on our measurements of cerebral autoregulation. We will also improve our experimental design by keeping consistent position between healthy controls and patients.

Given the vital role of cerebral blood flow and the importance of its regulation in response to changes in cerebral perfusion pressure, a safe, non-invasive, and reliable technique for the assessment of cerebral autoregulation would play an important role in clinical settings, and especially on patients with acute brain injuries (traumatic brain injury, aneurysmal subarachnoid hemorrhage, ischemic stroke, etc.). Changes in autoregulation and cerebrovascular reactivity is a known marker of injury severity and has the potential to guide therapeutics and patient care (60). The proposed technique, which is based on local optical measurement of cerebral hemodynamics that are driven by systemic variations in ABP, can be extended to spatial mapping of autoregulation. This is potentially important in the assessment of localized brain injuries associated with focal ischemic or hemorrhagic events. Even in cases where cerebral autoregulation is impaired in the entire brain, multiple local measurements may help reduce errors contributed by each individual measurement, thus enhancing reliability and minimizing inter-examiner variability. Future studies will aim to characterize the intra-subject variability of autoregulation measurements, which will serve as a basis for clinical studies on a larger patient population to assess the ability of this technique in determining the level of cerebral autoregulation in different pathological conditions.

## 5. Conclusion

In this study, we have shown that blood-pressure-induced oscillations in the cerebral concentrations of *O* and *D* measured by various FD-NIRS methods can be sensitive to cerebral autoregulation efficiency. These *O* and *D* measurements were translated into CBF dynamics by using the CHS model on healthy subjects, which is directly related to the measurements of dynamic cerebral autoregulation; we found these CBF dynamics to distinguish autoregulation efficiency between normocapnia and hypercapnia. Further investigations suggested that oscillations of *O*, and especially *D*, appeared to be more sensitive to the CBF dynamics than CBV dynamics. We have demonstrated the feasibility of measuring coherent *D* and ABP oscillations to assess autoregulation in the NCCU. Future studies will target a more complete characterization of the depth sensitivity of various data types of FD-NIRS measurements in heterogeneous tissue, especially in this protocol of systemic blood pressure oscillations, and applications to clinical scenarios with a larger sample size.

## Data Availability Statement

The raw data supporting the conclusions of this article will be made available by the authors, without undue reservation.

## Ethics Statement

The studies involving human participants were reviewed and approved by Tufts University Institutional Review Board. Written informed consent to participate in this study was provided by the participant or the participants' legal guardian/next of kin.

## Author Contributions

TP: study design, data collection on healthy subjects, analysis, and interpretation of healthy subject data. CF: analysis and interpretation of neurocritical care unit data. TP and CF: writing the original draft of the manuscript. KT: study design and data collection in the neurocritical care unit. SF: overall conceptualization, funding acquisition, and project supervision. GB, AS, and SF: data interpretation and critical review of the manuscript. JK and SB: clinical feedback and critical review of the manuscript. JK and XC: clinical study design and patient recruitment. All authors contributed to the article and approved the submitted version.

## Funding

This research was supported by the National Institutes of Health, Grant No. R01-NS095334. We also acknowledge support from the National Institutes of Health, Grant No. R21-EB020347, for the collection of data from patients in the neurological critical care unit. This study also received partial funding from the Neuroscience and Pain Research Unit at Pfizer, Inc. The funder was not involved in the study design, collection, analysis, interpretation of data, the writing of this article or the decision to submit it for publication.

## Conflict of Interest

The authors declare that the research was conducted in the absence of any commercial or financial relationships that could be construed as a potential conflict of interest.

## Publisher's Note

All claims expressed in this article are solely those of the authors and do not necessarily represent those of their affiliated organizations, or those of the publisher, the editors and the reviewers. Any product that may be evaluated in this article, or claim that may be made by its manufacturer, is not guaranteed or endorsed by the publisher.
