# Supplementary material for: Noninvasive Optical Measurements of Dynamic Cerebral Autoregulation by Inducing Oscillatory Cerebral Hemodynamics
Source: Front Neurol. 2021 Nov 16;12:745987. doi: 10.3389/fneur.2021.745987 (PMC8637213; doi:10.3389/fneur.2021.745987)
Supplement: Supplementary file 1 [file Data_Sheet_1.PDF]

frontiers

Supplementary Material

0.0.1 Hemodynamic Model to Determine the Relative Cerebral Blood Flow and Arterial Blood Pressure Dynamics

The two-dimensional phasor map of $\frac{\mathbf{cbf}(t,\omega)}{\mathbf{abp}(t,\omega)}$ (time (t) and frequency (ω)) could be obtained from the phasor ratio maps of $\frac{\mathbf{D}(t,\omega)}{\mathbf{ABP}(t,\omega)}$, $\frac{\mathbf{O}(t,\omega)}{\mathbf{ABP}(t,\omega)}$, and $\frac{\mathbf{T}(t,\omega)}{\mathbf{ABP}(t,\omega)}$ by using any of the following three approaches:

(1) When we have significantly high coherence for O versus ABP, D versus ABP, and T versus ABP within the region of interest of the induced oscillation:

$$\frac{\mathbf{cbf}(t,\omega)}{\mathbf{abp}(t,\omega)} = \frac{\frac{\mathbf{O}(t,\omega)}{\mathbf{ABP}(t,\omega)} - \frac{\mathbf{D}(t,\omega)}{\mathbf{ABP}(t,\omega)} - [(2S^{(a)}-1)\frac{\mathrm{CBV}_0^{(a)}}{\mathrm{CBV}_0^{(a)}+\mathrm{CBV}_0^{(v)}} + (2S^{(v)}-1)\frac{\mathrm{CBV}_0^{(v)}}{\mathrm{CBV}_0^{(a)}+\mathrm{CBV}_0^{(v)}}]\frac{\mathbf{T}(t,\omega)}{\mathbf{ABP}(t,\omega)}}{2\frac{T_0}{\mathrm{ABP}_0}[\frac{\langle S^{(c)}\rangle}{S^{(v)}}(\langle S^{(c)}\rangle - S^{(v)})\frac{\mathcal{F}^{(c)}\mathrm{CBV}_0^{(c)}}{\mathrm{CBV}_0}\mathcal{H}_{RC-LP}^{(c)}(\omega) + (S^{(a)}-S^{(v)})\frac{\mathrm{CBV}_0^{(v)}}{\mathrm{CBV}_0}\mathcal{H}_{G-LP}^{(v)}(\omega)]} \tag{S1}$$

(2) When we have significantly high coherence only for O versus ABP and T versus ABP within the region of interest of the induced oscillation:

$$\frac{\mathbf{cbf}(t,\omega)}{\mathbf{abp}(t,\omega)} = \frac{\frac{\mathbf{O}(t,\omega)}{\mathbf{ABP}(t,\omega)} - [S^{(a)}\frac{\mathrm{CBV}_0^{(a)}}{\mathrm{CBV}_0^{(a)}+\mathrm{CBV}_0^{(v)}} + S^{(v)}\frac{\mathrm{CBV}_0^{(v)}}{\mathrm{CBV}_0^{(a)}+\mathrm{CBV}_0^{(v)}}]\frac{\mathbf{T}(t,\omega)}{\mathbf{ABP}(t,\omega)}}{\frac{T_0}{\mathrm{ABP}_0}[\frac{\langle S^{(c)}\rangle}{S^{(v)}}(\langle S^{(c)}\rangle - S^{(v)})\frac{\mathcal{F}^{(c)}\mathrm{CBV}_0^{(c)}}{\mathrm{CBV}_0}\mathcal{H}_{RC-LP}^{(c)}(\omega) + (S^{(a)}-S^{(v)})\frac{\mathrm{CBV}_0^{(v)}}{\mathrm{CBV}_0}\mathcal{H}_{G-LP}^{(v)}(\omega)]} \tag{S2}$$

(3) When we have significantly high coherence only for D versus ABP and T versus ABP within the region of interest of the induced oscillation:

$$\frac{\mathbf{cbf}(t,\omega)}{\mathbf{abp}(t,\omega)} = -\frac{\frac{\mathbf{D}(t,\omega)}{\mathbf{ABP}(t,\omega)} - [(1-S^{(a)})\frac{\mathrm{CBV}_0^{(a)}}{\mathrm{CBV}_0^{(a)}+\mathrm{CBV}_0^{(v)}} + (1-S^{(v)})\frac{\mathrm{CBV}_0^{(v)}}{\mathrm{CBV}_0^{(a)}+\mathrm{CBV}_0^{(v)}}]\frac{\mathbf{T}(t,\omega)}{\mathbf{ABP}(t,\omega)}}{\frac{T_0}{\mathrm{ABP}_0}[\frac{\langle S^{(c)}\rangle}{S^{(v)}}(\langle S^{(c)}\rangle - S^{(v)})\frac{\mathcal{F}^{(c)}\mathrm{CBV}_0^{(c)}}{\mathrm{CBV}_0}\mathcal{H}_{RC-LP}^{(c)}(\omega) + (S^{(a)}-S^{(v)})\frac{\mathrm{CBV}_0^{(v)}}{\mathrm{CBV}_0}\mathcal{H}_{G-LP}^{(v)}(\omega)]} \tag{S3}$$

Here T_0 and ABP_0 are average values for absolute baseline total-hemoglobin concentration and arterial blood pressure, respectively. $S^{(a)}$, $\langle S^{(c)}\rangle$, and $S^{(v)}$ are the arterial, mean capillary, and venous saturation, respectively. $S^{(a)}$ is assumed as $S^{(a)} = 0.98$, $\langle S^{(c)}\rangle$ and $S^{(v)}$ can be calculated from $t^{(c)}$ and α as $\langle S^{(c)}\rangle = S^{(a)}(1-e^{-\alpha t^{(c)}})/(\alpha t^{(c)})$ and $S^{(v)} = S^{(a)}e^{-\alpha t^{(c)}}$, respectively. The complex transfer functions $\mathcal{H}_{RC-LP}^{(c)}(\omega)$ and $\mathcal{H}_{G-LP}^{(v)}(\omega)$ are associated with resistance-capacitance (RC) and Gaussian (G) low-pass filters as functions of ω, respectively, as described in detailed in Kainerstorfer et al. (2014).

REFERENCES

Kainerstorfer JM, Sassaroli A, Hallacoglu B, Pierro ML, Fantini S. Practical Steps for Applying a New Dynamic Model to Near-Infrared Spectroscopy Measurements of Hemodynamic Oscillations and Transient Changes: Implications for Cerebrovascular and Functional Brain Studies. *Acad Radiol* **21** (2014) 185–196. doi:10.1016/j.acra.2013.10.012.
